# MI_DenseNetCAM: A Novel Pan-Cancer Classification and Prediction Method Based on Mutual Information and Deep Learning Model

**DOI:** 10.3389/fgene.2021.670232

**Published:** 2021-06-03

**Authors:** Jianlin Wang, Xuebing Dai, Huimin Luo, Chaokun Yan, Ge Zhang, Junwei Luo

**Affiliations:** ^1^School of Computer and Information Engineering, Henan University, Kaifeng, China; ^2^College of Computer Science and Technology, Henan Polytechnic University, Jiaozuo, China

**Keywords:** pan-cancer, cancer classification, DenseNet, guided grad-CAM algorithm, RNA-seq data

## Abstract

The Pan-Cancer Atlas consists of original sequencing data from various sources, provides the opportunity to perform systematic studies on the commonalities and differences between diverse cancers. The analysis for the pan-cancer dataset could help researchers to identify the key factors that could trigger cancer. In this paper, we present a novel pan-cancer classification method, referred to MI_DenseNetCAM, to identify a set of genes that can differentiate all tumor types accurately. First, the Mutual Information (MI) was utilized to eliminate noise and redundancy from the pan-cancer datasets. Then, the gene data was further converted to 2D images. Next, the DenseNet model was adopted as a classifier and the Guided Grad-CAM algorithm was applied to identify the key genes. Extensive experimental results on the public RNA-seq data sets with 33 different tumor types show that our method outperforms the other state-of-the-art classification methods. Moreover, gene analysis further demonstrated that the genes selected by our method were related to the corresponding tumor types.

## 1. Introduction

Cancer, known as the “the king of the diseases,” is a serious threat to human health. In 2020, 1,806,590 new cancer cases and 606,520 cancer deaths are projected to occur in the United States (Siegel et al., 2020). Cancer accurate prediction in the early stage is a challenging subject that has drawn worldwide concern due to the high morbidity and mortality of cancer (Kourou et al., 2015). However, the existing medical equipment and clinical symptoms are not sensitive to the changes at the molecular level, and it is difficult to make early diagnosis for potential patients. Some potential patients cancer may be advanced when they are first diagnosed (Sakri et al., 2018), resulting in increased mortality from cancer. If cancer can be detected early and treated appropriately, the survival time of patients will be greatly increased. Therefore, identifying a set of genes that can characterize the type and stage of cancer is the key to effective treatment. These genes may serve as biomarkers to efficiently diagnose diseases and accurately classify cancer types. Furthermore, since The Cancer Genome Atlas (TCGA) project was launched, TCGA project has so far generated a pan-cancer atlas of 33 types of cancer. Therefore, extensive studies about pan-cancer have been researched, among which pan-cancer classification is an important perspective.

In recent years, advances in sequencing technology have led to a significant decrease in the cost of accumulating biological data. A large amount of biological data laid an important foundation for researchers to identify some key cancer biomarkers and enable accurate cancer classification prediction in the early stage. However, the tough challenges also come from the characteristics of these data (i.e., high dimensionality, severely limited samples and containing a large portion of irrelevant genes), which hinders the rapid and accurate cancer classification and prediction (Saeys et al., 2007). In order to solve this problem, feature selection techniques can be applied to analyze the possible cancer-causing genes from massive cancer gene data. The feature selection aims to represent high-dimensional data with fewer features while improves the prediction accuracy of classification models. In general, feature selection can be categorized into two types: filter methods, wrapper methods (Huang et al., 2007). Usually, filter methods have much less computational complexity compared with other methods. Some filter methods, such as MI, IG (Martín-Valdivia et al., 2008), Relief (Urbanowicz et al., 2018), have been applied to data analysis for gene expression data well.

However, most traditional tumor classification studies only focus on the same tumor type, the heterogeneity among different tumor types is usually neglected (Lawrence et al., 2013; Lyu and Haque, 2018). Tumor heterogeneity is reflected in the obvious differences between different tumor cells at the molecular level of genomic, transcriptomic, proteome and so on. Therefore, in order to understand and capture the commonalities and differences between diverse cancers, TCGA later launched the Pan-Cancer analysis project (Weinstein et al., 2013). Pan-cancer analysis is a study that integrates multiple tumor types. In recent years, the research and analysis of pan-cancer have been increasing gradually, and people hope to find the genes related to tumors so as to accurately predict the type of cancer. It has been suggested that specifications of therapies according to tumor types differentiated may maximize the efficacy of the patients (Golub et al., 1999; Alizadeh et al., 2000; Van't Veer et al., 2002). At present, there have been many studies (Kourou et al., 2015; Li et al., 2017) using machine learning (ML) algorithms to analyze pan-cancer data sets and demonstrate its effectiveness in cancer classification and prediction. For example, Li et al. proposed a GA/KNN method to classify 9,096 samples from 31 different tumor types and obtained a set of genes that could correctly classify 90% of the samples. Deep learning has made unprecedented breakthroughs in various classification tasks recently and has been widely applied due to its excellent classification performance. A strength of deep learning is its ability to learn end to end, automatically discovering multiple levels of representation to achieve a prediction task (Wainberg et al., 2018).

In the study, a deep learning approach, MI_DenseNetCAM was proposed to classify 33 different types of tumors based on high-dimensional RNA-Seq gene expression data. Then, the Guided grad-CAM algorithm was used to identify the key genes that played an important role in the classification process. We evaluated the method with performance metrics such as recall, precision and F1 score, and the results demonstrate that the proposed method takes full advantage of the information in the pan-cancer data sets and achieved an overall test accuracy of 96.81%. Compared with the existing methods, our proposed method provides superior performance in the classification accuracy of 33 tumor types. The main contributions of this paper can be summarized as follows:

- For the noise and redundancy of the pan-cancer data sets, the Min-Max normalization and MI was adopted to preprocess the data, which can screen out the highly correlated genes to improve the performance of the classification model. Moreover, we evaluated the impact of different data preprocessing strategies on the classification performance.
- For the pan-cancer data set, the DenseNet model was utilized as a classifier to classify and predict tumor types. Compared with other classifiers, the DenseNet model achieved better performance whilst requiring fewer parameters and computation cost.
- Extensive experiments and analyses have been carried out on the pan-cancer data set in terms of evaluation indicators, and the experimental results demonstrate that our proposed method is very promising. Some of the genes identified by our method have already been verified.

The remainder of this paper is organized as follows: In section 2, we review related works. In section 3, the detailed implementation of the proposed pan-cancer classification method is elaborated. We described the experimental results and analysis in section 4. Finally, we summarize the paper and discuss the future works in section 5.

## 2. Related Work

The goal of the pan-cancer analysis was to assemble data from the separate disease projects to build a data set spanning multiple tumor types (Weinstein et al., 2013). Through the analysis and interpretation of these data to find the commonalities and differences across various tumor types. At present, many machine learning and deep learning methods have been applied to the analysis of pan-cancer data. Next, we conduct a review of the latest studies in the field of pan-cancer analysis.

Hsu and Si (2018) focused on using machine learning (ML) to build a reliable classification model which can recognize 33 types of cancer patients. They applied five ML algorithms, namely decision tree (DT), k nearest neighbor (kNN), linear support vector machine (linear SVM), polynomial support vector machine (ploy SVM), and artificial neural network (ANN) to analyze the data set of pan-cancer. The results show that linear SVM with a 95.8% accuracy rate is the best classifier among the five classification algorithms.

Kang et al. (2019) proposed a new method for the classification of multiple tumor types by using relaxed Lasso selection feature subsets and an improved support vector machine (GenSVM) as the classifier. GenSVM is a general multiclass support vector machine, which compared with the other three classifiers (KNN, L_1_logreg, L_2_logreg) on the four multi-class datasets, the experimental results showed that GenSVM has better generality, flexibility and achieve higher classification accuracy with fewer features in multi-classification problems.

Li et al. (2017) undertook the development of a pan-cancer atlas to recognize 9,096 TCGA tumor samples representing 31 tumor types. They applied k-nearest neighbors (KNN) to classify 31 different types of tumor, and embedded genetic algorithm to improve the accuracy of the KNN classifier. This method achieved an accuracy of 90% across 31 tumor types.

In recent years, the deep learning (DL) method was also used to classify and identify cancer types. In paper (Danaee et al., 2017) the author used a stacked auto-encoder first to extract high-level features from the expression values and then input these features into a single layer ANN network to decide whether the sample is a tumor or not. The accuracy of using such a method reached 94%. However, as to the multi-classification problem, because this method has more complicated network structure and parameter setting, in order to save time cost, the author only conducted the experiments on breast cancer.

Khalifa et al. (2020) introduced a novel optimized deep learning approach based on binary particle swarm optimization with decision tree (BPSO-DT) and CNN to classify different types of tumor. The results showed that the proposed method achieved an overall testing accuracy of 96.6%. However, they classified only five different tumor types (KIRC, BRCA, LUSC, LUAD, and UCEC), and did not analyze all the pan-cancer data sets.

Lyu and Haque (2018) designed a new method that embedded the high dimensional RNA-Seq data into 2-D images and used a CNN to make classification of the 33 tumor types. This method achieved 95.59% accuracy for all 33 tumor types. However, the method proposed by Lyu et al. failed to achieve good classification performance on tumor datasets with small samples, which increases the risk of overfitting.

## 3. Materials and Methods

**Figure 1:**
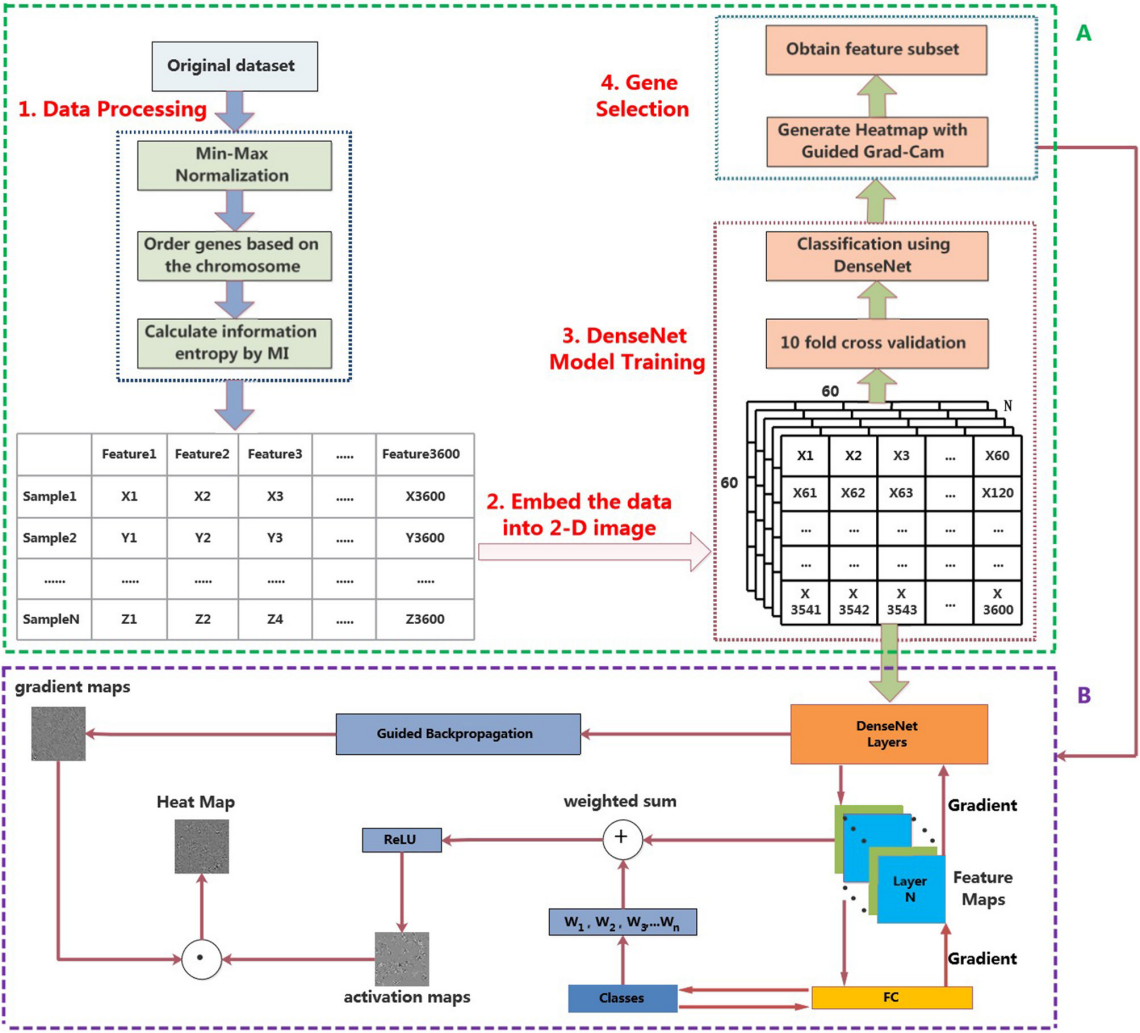
The workflow of MI_DenseNetCAM. **(A)** Cancer classification and prediction through MI and deep learning combined analysis from pan-cancer datasets. **(B)** Principle diagram of the Guided Grad-Cam algorithm.

In this section, a novel framework for the classification of pan-cancer, referred to MI_DenseNetCAM has been proposed. First, we preprocess the original data set, and then embed the data into a 2-D image. Then, we train a DenseNet model with the generated images. Next, the trained model and Guided Grad-Cam algorithm are applied to generate the heat map. Furthermore, some important genes can be obtained. The workflow of the proposed method is shown in Figure 1A.

### 3.1. Datasets

We conducted experiments to evaluate the proposed method on the RNA-seq data sets of 33 types of cancers. RNA-seq, also known as transcriptomic sequencing, can accurately analyze gene expression differences and gene structure variations, and reveal specific biological processes and molecular mechanisms in the process of disease occurrence. Therefore, we use the normalized-level3 RNA-seq gene expression data to construct our experiment dataset. The datasets are available for download from http://gdac.broadinstitute.org/. These data sets, which contain 33 different tumor types. The data for each type of tumor is high-dimensional, with 20,531 columns. **Table 3** gives a detailed description of the number of samples and genes in these datasets.

### 3.2. Data Preprocessing

Firstly, data from 33 different tumor types are collected and integrated, and then the genes in the data set are compared with the annotation files (downloaded from NCBI), so as to screen out the genes that did not exist in the annotation files. About 1,000 genes were not found in the annotation file, therefore, these 1,000 genes and corresponding expression levels need to be removed from the data set. Secondly, genes are ordered based on the chromosome number because adjacent genes are more likely to interact with each other. Thirdly, the data set is normalized by Min-Max normalization, which scales the data to a small interval, thus leads to get the solution quickly. The Min-Max normalization is defined by Equation (1).

(1) $$\begin{array}{l} y=\frac{X-X_{min}}{X_{max}-X_{min}} \end{array}$$

Where X represents a column of data in the pan-cancer data set, X_*min*_ and X_*max*_ represent the minimum and maximum values in a column of data.

After normalization of gene data, we further adopted Mutual Information (MI) to calculate the correlation between the gene and the label to decide whether to select the gene. MI is a feature ranking approach based on information entropy (Kraskov et al., 2004; Martín-Valdivia et al., 2008). In the domain of feature selection, Sharmin et al. (2019) used MI as a metric to measure the degree of correlation between features and category labels. The more mutual information between the two, the more important this feature is. The mutual information between two random variables *X* and *Y* is as follows:

(2) $$\begin{array}{l} I\left(X,Y\right)=\underset{x,y}{\sum }P\left(x,y\right)log\frac{P\left(x,y\right)}{P\left(x\right)P\left(y\right)} \end{array}$$

Where, *P(x,y)* represents the joint probabilistic mass function, *P(x)* and *P(y)* represent edge probability density functions. The closer the relationship between *X* and *Y* is, the greater the value of *I(X, Y)* will be. If the two variables are independent, the value of *I(X, Y)* is 0.

When mutual information is applied to feature selection, then random variable *X* represents the feature and random variable *Y* represents the label, the value of *I(X, Y)* represents the correlation between the ith feature and the label. The greater the value, the greater the correlation between the feature and the label, and vice versa. Therefore, we can sort features in terms of the information entropy by MI method and select important features.

After mutual information, the number of genes was further reduced to N. Through the subsequent experiment of different N values, N is set to 3,600. Then, convert the data corresponding to the selected important features into an image format. The data from each sample are successively put into each pixel of the image in order to reconstruct the data from a 1-D array into a 2-D image. In other words, the array with the shape of 3600*1 is turned into a two-dimensional image with the shape of 60*60, and the data needs to be normalized to [0,255]. The result of this step is to generate images that correspond to the samples in the dataset. The resulting 2-D images will be used to train the DenseNet model.

### 3.3. Model Training

**Figure 2:**
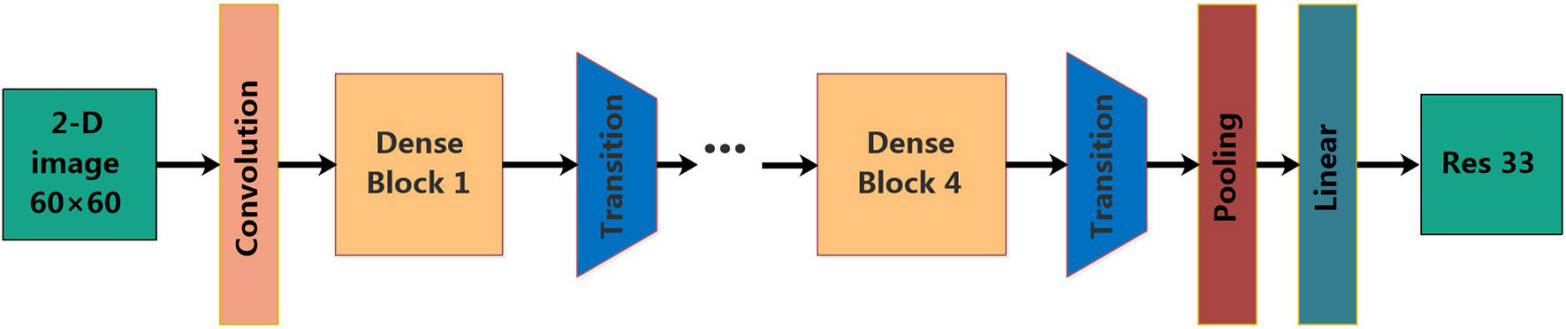
The structure of the DenseNet.

Deep neural models based on Convolutional Neural Network (CNN) have enabled unprecedented breakthroughs in a variety of image classification tasks, some famous architectures such as Resnet (He et al., 2016a) and inception (Szegedy et al., 2015) have excellent performance. In the Imagenet (Deng et al., 2009) challenge, CNN achieved a significant classification accuracy margin over classical machine learning methods. However, with the increase of layers, the traditional neural network will encounter a series of problems, such as gradient vanishing, feature reuse decreasing, parameter number increasing significantly, longer training time and classification accuracy decreasing (He et al., 2016b). In order to solve these problems, Huang et al. proposed a new method, DenseNet (Huang et al., 2017), which is a convolutional neural network with dense connections. Dense Net connects all layers directly to each other to ensure the maximum information flow between each layer in the network, in other words, the input of any next layer in the network is the superposition of the output of all previous layers. In this way, each layer can access the gradient directly from the loss function and the original input signal, yielding models that are easy to train and highly parameter efficient. Further, the dense connections have a regularizing effect, which reduces the risk of overfitting for small sample training tasks (Huang et al., 2017). The structure of the DenseNet is as shown in Figure 2.

The DenseNet model consists of four Dense Blocks, and each Dense Block is composed of batch normalization layer (BN) + ReLU + 1 × 1 convolutional layer (Conv 1 × 1) + BN + ReLU + Conv 3 × 3. The layers between two adjacent blocks are referred to as transition layers, which are composed of BN + ReLU + Conv 1 × 1 + Average Pooling 2 × 2. Pooling denotes the global average pool and Linear denotes the fully connected layer.

The optimizer plays an extremely significant role in deep learning training. It is used to update the weight parameters in the training process, which is related to whether the training can converge quickly and achieve high accuracy. In this paper, we use the Adam optimization algorithm. Compared with the traditional stochastic gradient descent algorithm, the advantage of the Adam algorithm is that it can design independent adaptive learning rates for different parameters, so as to obtain a higher training effect. For the classification task, cross entropy is generally used as the loss function. Moreover, In order to get a better training effect and ensure the robustness of the classification, make full use of the generated 2-D images and obtain reliable and stable models, we use 10-fold cross validation to evaluate the quality of the model during the training of DenseNet.

### 3.4. Screen Out Important Genes

After the DenseNet model is trained, the important genes can be obtained through two stages. First, the Guided Grad-Cam algorithm can be applied to generate heat maps, it can locate the regions related to categories in the image, indicating why the convolutional neural network is classified in this way. Then, match the high-intensity pixels in the heat map with the gene names in the original data set to obtain the important genes that contribute more to the classification.

The Guided Grad-Cam algorithm provides a technique for visual interpretation of how the convolutional neural network model makes decisions. The detailed procedure for generating heat map through the Guided Grad-Cam algorithm is as follows.

- Step1 Obtain Gradient maps
  First, the Guided backpropagation algorithm is used to calculate the gradient of the convolutional layer's feature value relative to the input layer, so as to obtain the feature gradient maps.
- Step2 Obtain Activation maps
  After feature extraction of the original image through the convolutional layer and the pooling layer, the convolutional neural network output a set of feature maps. A pixel in the feature map corresponds to a region in the original image. If the product of pixel value and weight in the feature map is >0, CNN believes that this region in the original image has features related to categories. The Guided Grad-Cam algorithm calculates the average gradient of each feature map relative to the classification probability to obtain a set of weights. After calculating the weights of all the feature maps, the weighted sum with the feature maps can be used to obtain the activation maps. Finally, the activation maps are processed using the ReLU activation function, retaining only the features of the activation maps that are useful for the category. If you do not add the ReLU activation function, you will bring in pixels belonging to other categories, which will affect the interpretation.
- Step3 Obtain Heat maps
  Superposition the gradient maps and activation maps to obtain the heat map for visualization of the convolutional neural network. The heat map shows the extent to which the pixel at the corresponding position in the original image affects the classification result. The overall structure of the Guided Grad-Cam algorithm is shown in Figure 1B.

Based on the DenseNet model and the Guided Grad-Cam algorithm, we can obtain heat maps with high resolution and category discriminability for displaying the importance of genes. Since the Guided Grad-Cam algorithm generates a heat map for each input image, in order to avoid the influence of noise on the experimental results, we averaged all the heat maps. In addition, the intensity of the pixel value in the heat map represents the influence of the pixel at the corresponding position in the original image on the classification result, so the gene corresponding to the position with the largest pixel value in the heat map is an important feature. In other words, the higher the pixel value and the higher the intensity in the heat map, the greater the contribution of these pixels to the final classification, that is their existence affects the classification most. So, important genes can be realized by looking for points with high pixel intensity in the heat map. The specific methods to achieve the following.

When the gene expression data is converted into 2D images, the corresponding expression values of each gene are sequentially mapped to the pixels in the images, as shown in Figure 1A. In order to screen out the important genes, firstly, we can convert the pixel value in the heat map into a 1D array based on their original order. Then, find out the index corresponding to the maximum pixel value, the corresponding gene name can be found from the original data according to the index value.

## 4. Experiments and Results

In order to verify the performance of our method, we compared it with the other four state-of-the-art methods, namely MI_KNN, Relaxed Lasso and generalized multi-class support vector machine (rL-GenSVM) (Kang et al., 2019), Variance_CNN (Var_CNN) (Lyu and Haque, 2018) and ExtraTrees-SVM (ET-SVM) (Hsu and Si, 2018). These methods can realize multiple classification and feature selection of tumors, and have achieved a good classification effect in biomedical data. Our experiment consists of two parts. Firstly, we conducted an experiment on the classification performance of the model to verify that our method could achieve better classification effect in 33 different tumor types. Then, we evaluated the corresponding classification errors of 5 methods when selecting different gene numbers, indicating that our method can obtain a better subset of features. All experiments were executed on a computer server with Windows 7 operating system, Intel Core(TM) i7-10700 CPU (2.9 GHz), 32 GB RAM, 8 GB Nvidia GeForce RTX 2080 SUPER, using Python language.

### 4.1. Evaluation Metrics

Since pan-cancer classification is a multi-classification problem, we use accuracy to measure the performance. At the same time, to evaluate the performance of the proposed architecture, more performance measures need to be investigated in this research. There are also precision, recall and F1Score (Goutte and Gaussier, 2005) to measure performance in a classification problem. The evaluation indicator is defined as follows.

Accuracy: the ratio of the number of samples correctly classified by the classifier to the total number of samples for a given test data set. The calculation formula is shown below.

(3) $$\begin{array}{l} Acc=\frac{TP+TN}{TP+FP+FN+TN} \end{array}$$

Precision: It represents how many of the samples predicted to be positive are correct. The calculation formula is shown below.

(4) $$\begin{array}{l} P=\frac{TP}{TP+FP} \end{array}$$

Recall rate: This is how much of the positive sample was predicted correctly. The calculation formula is shown below.

(5) $$\begin{array}{l} R=\frac{TP}{TP+FN} \end{array}$$

F1-Score: The harmonic mean of the precision rate and recall rate. It is a combination of precision rate and recall rate. The calculation formula is shown below.

(6) $$\begin{array}{l} F1Score=\frac{2PR}{P+R} \end{array}$$

This research uses the 10-fold cross-validation to calculate accuracy, precision, recall and F1Score.

### 4.2. Parameters Settings

In this section, the parameter values of all methods are given in Table 1. For Var_CNN, rL-GenSVM and ET-SVM, we chose parameter values according to relevant literature (Hsu and Si, 2018; Lyu and Haque, 2018; Kang et al., 2019). For the proposed method, the values of growth_rate and compression_factor are set to 16, 0.5, respectively, which has been analyzed and evaluated in previous studies (Huang et al., 2017). Based on our experimental analysis, the value of image_dimension is set to 60. Similar to Var_CNN, we take the same value for learning_rate and num_epochs.

**Table 1:** Parameter settings.

**Algorithm**	**Parameter**
MI_DenseNetCAM	learning_rate = 0.0001, num_epochs=200, batch_size = 32, growth_rate = 16, compression_factor = 0.5, image_dimension = 60
MI_KNN	n_neighbors = 5
Var_CNN	learning_rate = 0.0001, num_epochs = 200, batch_size = 500
rL-GenSVM	phi = 1/3, *p* = 1, kernel = “rbf,” epsilon = 1e-3, lambda = 1e-9, gama = 1e-8, kappa = 2
ET-SVM	C = 0.004, kernel = “linear,” decision_function_shape = “ovo,” gama = 1

### 4.3. Comparison With Other Methods

In this section, we compare the average accuracy, precision, recall and F1-score of MI_KNN, Var_CNN, rL-GenSVM and ET-SVM algorithm. The overall classification results of these methods on 33 tumor types are shown in Table 2.

**Table 2:** The experimental results of five methods.

**Method**	**Accuracy**	**Precision**	**Recall**	**F1-Score**
MI_DenseNetCAM	96.81%	96.89%	96.81%	96.85%
MI_KNN	92.61%	92.46%	92.61%	92.40%
Var_CNN	95.59%	95.54%	95.59%	95.43%
rL-GenSVM	87.29%	87.73%	87.29%	86.91%
ET-SVM	90.73%	90.22%	90.73%	89.99%

It can be seen from Table 2, in terms of accuracy, precision, recall and f1-score, the proposed method MI_DenseNetCAM performs best on the pan-cancer datasets. At the same time, a comparison of accuracy as to each class is shown in Table 3. From the previous two experiments, we can see that DenseNet has higher accuracy when using the same preprocessing algorithm. Then, we compared the method with that in the literature (Lyu and Haque, 2018), which makes a similar contribution to our study. Although the overall classification result is only 1.22% higher than Var_CNN algorithm, in terms of the specific accuracy of each class, our method performs better. Especially in ACC, CESC, CHOL, ESCA, MESO and PAAD datasets. Meanwhile, compared with Var_CNN algorithm, our method also has better performance in small sample datasets. The accuracy of our method is 100, 75, and 99% respectively for dataset ACC, CHOL and MESO, and the results are higher than the accuracy obtained by Var_CNN. whose accuracy is 95, 56, and 94% respectively. Since the Guided Grad-CAM algorithm generates heat maps based on the prediction results of each class, the higher the precision in each class, the more likely it is to use heat maps to obtain the optimal subset of features. Moreover, our method requires fewer parameters and uses parameters more efficiently, which can be reflected in the size of the model. our model only uses 13.9 M parameters to achieve an accuracy of 96.81%, while the model of Var_CNN uses 295 M parameters to achieve an accuracy of 95.59%. To achieve a similar level of accuracy, our method only requires around 1/21 of the parameters of Var_CNN. Finally, we compared some of the methods introduced in the related work, and the results show that our method also shows superior performance.

**Table 3:** Benchmark datasets.

**Tumor type**	**Cohort**	**Instances**	**MI_Dense NetCAM**	**MI_KNN**	**Var_CNN**	**rL-GenSVM**	**ET-SVM**
Adrenocortical carcinoma	ACC	79	1	0.95	0.95	0.63	0.92
Bladder urothelial carcinoma	BLCA	408	0.98	0.87	0.97	0.53	0.78
Breast invasive carcinoma	BRCA	1093	0.99	0.99	0.99	0.92	0.99
Cervical and endocervical cancers	CESC	304	0.95	0.88	0.93	0.65	0.86
Cholangiocarcinoma	CHOL	36	0.75	0.58	0.56	0.40	0
Colon adenocarcinoma	COAD	457	0.95	0.99	0.95	0.82	0.98
Lymphoid Neoplasm Diffuse Large B-cell Lymphoma	DLBC	48	1	1	1	1	1
Esophageal carcinoma	ESCA	184	0.85	0.69	0.77	0.50	0.45
Glioblastoma multiforme	GBM	160	0.95	0.92	0.94	0.83	0.81
Head and Neck squamous cell carcinoma	HNSC	520	0.99	0.95	0.98	0.96	0.94
Kidney Chromophobe	KICH	66	0.89	0.75	0.87	0.80	0.64
Kidney renal clear cell carcinoma	KIRC	533	0.94	0.93	0.95	0.89	0.95
Kidney renal papillary cell carcinoma	KIRP	290	0.94	0.86	0.93	0.82	0.83
Acute Myeloid Leukemia	LAML	179	1	1	1	1	1
Brain Lower Grade Glioma	LGG	516	1	0.95	0.98	0.96	0.98
Liver hepatocellular carcinoma	LIHC	371	0.97	0.96	0.97	0.91	0.96
Lung adenocarcinoma	LUAD	515	0.95	0.91	0.95	0.91	0.96
Lung squamous cell carcinoma	LUSC	501	0.93	0.85	0.91	0.84	0.82
Mesothelioma	MESO	87	0.99	0.95	0.94	0.89	0.62
Ovarian serous cystadenocarcinoma	OV	304	1	0.98	0.99	1	1
Pancreatic adenocarcinoma	PAAD	178	1	0.97	0.97	0.95	0.64
Pheochromocytoma and Paraganglioma	PCPG	179	1	0.99	1	0.95	0.96
Prostate adenocarcinoma	PRAD	497	0.99	1	1	0.96	0.99
Rectum adenocarcinoma	READ	166	0	0	0.35	0	0
Sarcoma	SARC	259	0.98	0.95	0.97	0.74	0.98
Skin Cutaneous Melanoma	SKCM	469	0.98	0.97	0.98	1	0.96
Stomach adenocarcinoma	STAD	415	0.96	0.90	0.96	0.93	0.98
Testicular Germ Cell Tumors	TGCT	150	1	0.99	0.99	1	0.83
Thyroid carcinoma	THCA	501	1	1	1	1	0.99
Thymoma	THYM	120	1	0.98	0.99	1	0.91
Uterine Corpus Endometrial Carcinoma	UCEC	545	0.95	0.92	0.96	0.95	0.78
Uterine Carcinosarcoma	UCS	57	0.83	0.72	0.81	0.83	0
Uveal Melanoma	UVM	80	1	1	0.99	1	1

Next, we further evaluated the performance of our proposed method. First, we conducted experiments on the DenseNet model without any preprocessing. In terms of Accuracy, Precision, Recall and F1-Score, the DenseNet model without preprocessing can achieve 93.90, 94.03, 93.90, and 93.89% respectively. Then, we evaluated the effects of different preprocess strategies (Variance, Chi2, *F*-Test, MI) on the classification performance. The experimental results are shown in Table 4. It can be seen from Table 4 that preprocessing based on ML can further improve the accuracy of the classifier. Meanwhile, compared with other methods, MI has better performance on all indicators.

**Table 4:** The performance evaluation results of different preprocess strategies.

**Method**	**Accuracy**	**Precision**	**Recall**	**F1-Score**
Var_DenseNet	94.46%	94.62%	94.46%	94.37%
Chi2_DenseNet	95.42%	95.54%	95.42%	95.40%
FTest_DenseNet	95.03%	95.20%	95.03%	95.01%
MI_DenseNetCAM	96.81%	96.89%	96.81%	96.85%

### 4.4. The Impact of Classifiers on Performance

To further evaluate the impact of different classifiers on the performance of our method, four classifiers, namely KNN, CNN, SVM and DenseNet, are selected to conduct experiments on the pan-cancer data set. The experimental results are shown in Table 5. Compared with the other three classifiers, the DenseNet model shows better performance in terms of different evaluation indicators.

**Table 5:** The performance evaluation results of four different classifiers.

**Classifiers**	**Accuracy**	**Precision**	**Recall**	**F1-Score**
MI_KNN	92.61%	92.46%	92.61%	92.40%
MI_CNN	94.30%	94.37%	94.30%	94.28%
MI_SVM	91.53%	91.67%	91.53%	90.97%
MI_DenseNetCAM	96.81%	96.89%	96.81%	96.85%

### 4.5. The Impact of Image Dimensions on Performance

To further evaluate the impact of image dimension on the performance of the proposed method, in this section, various image dimensions are adopted to conduct experiments. The experimental results are shown in Table 6. As can be seen from Table 6, MI_DenseNetCAM achieves the best performance when the image dimension is set to 60.

**Table 6:** The performance evaluation results of different image dimensions.

**Dimensions**	**Accuracy**	**Precision**	**Recall**	**F1-Score**
30 * 30	93.60%	93.54%	93.60%	93.46%
50 * 50	95.03%	94.82%	95.03%	94.85%
60 * 60	96.81%	96.89%	96.81%	96.85%
70 * 70	95.22%	95.41%	95.22%	95.23%
90 * 90	94.17%	94.18%	94.17%	94.07%
110 * 110	92.93%	93.18%	92.93%	92.81%
130 * 130	93.41%	93.88%	93.41%	93.34%

### 4.6. Evaluate Important Genes

**Figure 3:**
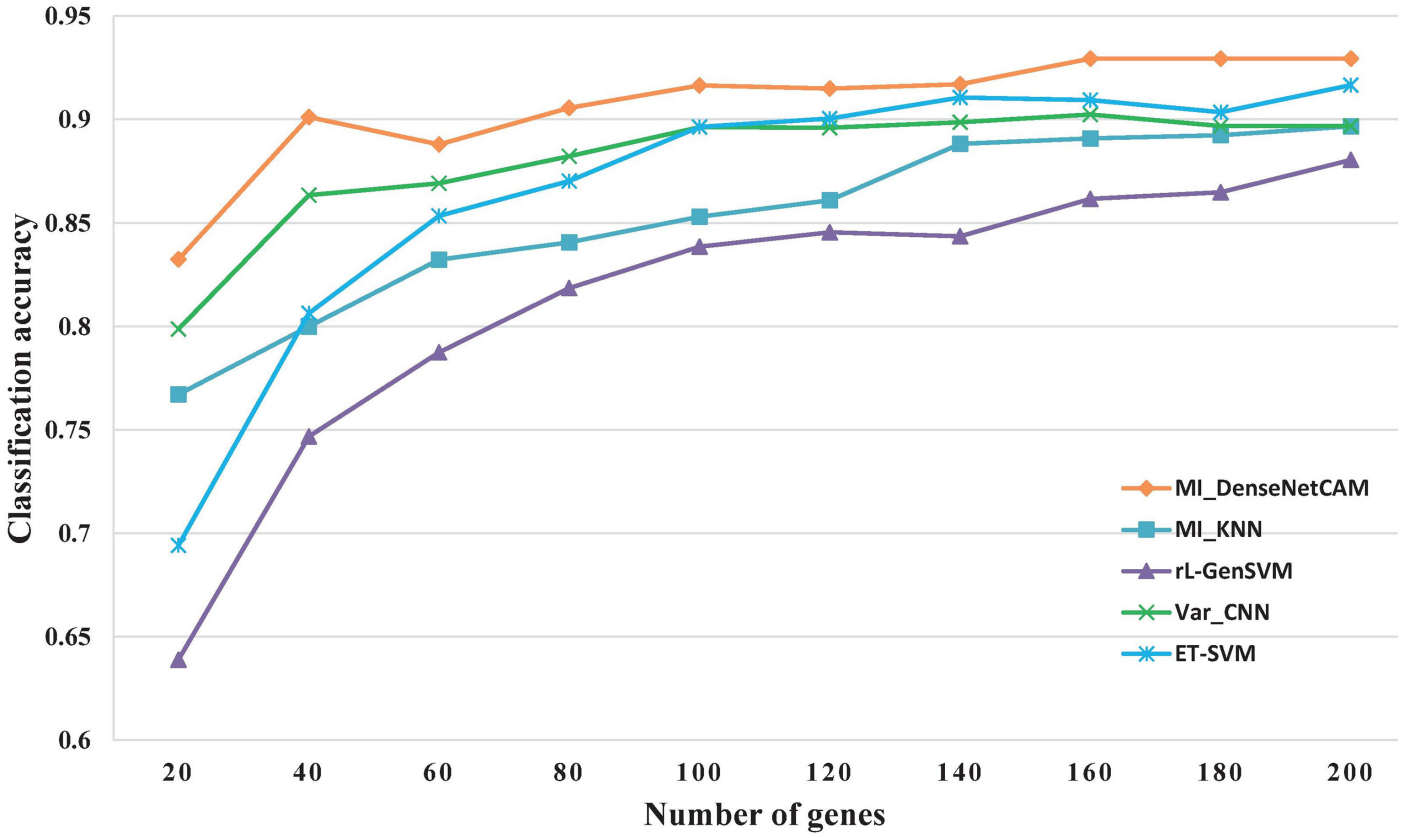
The Classification accuracy of different gene numbers.

However, discovering some key genes quickly will reduce the workload of following biological experiments, and help the rapid disease diagnosis. As a result, it is meaningful to obtain small gene sets with high classification accuracy. For the issue, we further evaluated the classification performance for all methods based on small scale genes ranges from 20 to 200. The experimental results are shown in Figure 3. The results show that the proposed MI_DenseNetCAM is superior to other methods. It can achieve 83.24% accuracy only using 20 genes.

As can be seen from Figure 3, in terms of classification accuracy, MI_DenseNetCAM has the best effect, which is obviously superior to the other four methods, while the rL-GenSVM method has the worst effect, with the accuracy can only be up to 86%. For the other three methods of MI_KNN, ET-SVM and Var_CNN, although their performance is unsatisfactory in the case of a small number of genes, their accuracy is improved with the increase of the number of genes. Compared with the other four methods, MI_DenseNetCAM usually requires fewer genes under the condition of the same precision. For example, with the highest accuracy of 86% of rL-GenSVM as the baseline, we compared the number of genes needed to achieve this accuracy with other methods. MI_DenseNetCAM only requires 25 genes to achieve this accuracy, while ET-SVM requires 70 genes, Var_CNN requires 85 genes, and MI_KNN requires the most. It requires 130 genes. In addition, from Figure 3, it is obvious that MI_DenseNetCAM can obtain higher prediction accuracy than the other four methods when dealing with the same number of genes. Therefore, both in terms of the number of genes and accuracy, our method can achieve better performance.

### 4.7. Gene Analysis

In this section, we conduct further analysis and verify the selected genes by the proposed method. These genes selected by our proposed method are lists in Table 7.

**Table 7:** Selected genes.

**Number of genes**	**The name of the gene**
40	GSTA1, C4A, COL3A1, PABPC1, COL1A1, KRT13, S100A6, SERPINA1, FGA, MUC2, COL1A2, APOE, KRT5, MALAT1, GFAP, TUBA1A, KRT14, KLK1, ATP1A1, RGS5, SPP1, CLU, S100A9, TF, APOC1, MUC1, ADAM6, SFTPA2, BCAM, TTR, CHGA, SCG2, FASN, PDLIM5, LGALS4, CA2, MYH11, SILV, PGC, TG

We selected 40 genes for further analysis, because it can be seen from Figure 3 that the accuracy of 40 genes was already very high, and the accuracy did not improve significantly with the increase of the number of genes. Next, the KEGG pathway analysis results for 40 genes are obtained using the David website (https://david.ncifcrf.gov/), trying to find out if significantly enriched pathways are related to the tumor. Pathway analyses showed those genes were significantly enriched in 31 KEGG pathways [Log10(P) < −2 or *P* < 0.01], which mainly involved in complement activation, cell projection, cellular response, cellular activities such as adhesion, migration, differentiation, proliferation, and apoptosis (Table 8). Some of these pathways are already involved in cancer development. For example, hsa04610 might contribute to the progression of bladder cancer (Liu et al., 2020). The hsa05133 pathway is related to the hsa04610 pathway, so it also promotes bladder cancer formation. In the hsa04611 pathway, cancer cells migrate to the vasculature and interact with platelets, causing inflammation and promoting mesothelioma growth (Jurasz et al., 2004; Sekido, 2013). The hsa04512 pathway interaction is involved in six critical cancer hallmarks (Pickup et al., 2014). So, the related genes in these pathways can then be viewed as tumor specific biomarkers.

**Table 8:** The KEGG pathway analysis.

**KEGG Pathways**	**Description**	***P*-Value**	**Genes**
hsa04610	Complement and coagulation cascades	9.50E-09	C3,CLU,C4A,FGA,SERPINA1
hsa05133	Pertussis	6.40E-07	C3,CALML3,SFTPA2,C4A
hsa04974	Protein digestion and absorption	1.22E-06	COL3A1,COL1A2,ATP1A1,COL1A1
hsa05146	Amoebiasis	1.50E-06	COL3A1,MUC2,COL1A2,COL1A1
hsa04611	Platelet activation	4.19E-06	COL3A1,COL1A2,FGA,COL1A1
hsa04918	Thyroid hormone synthesis	3.80E-05	TG,ATP1A1,TTR
hsa04971	Gastric acid secretion	3.95E-05	CALML3,CA2,ATP1A1
hsa04933	AGE-RAGE signaling pathway in diabetic complications	9.05E-05	COL3A1,COL1A2,COL1A1
hsa04926	Relaxin signaling pathway	1.93E-04	COL3A1,COL1A2,COL1A1
hsa04964	Proximal tubule bicarbonate reclamation	2.02E-04	CA2,ATP1A1
hsa04915	Estrogen signaling pathway	2.29E-04	CALML3,KRT14,KRT13
hsa04145	Phagosome	3.02E-04	C3,TUBA1A,SFTPA2
hsa04979	Cholesterol metabolism	8.78E-04	APOE,APOC1
hsa04961	Endocrine and other factor-regulated calcium reabsorption	8.78E-04	KLK1,ATP1A1
hsa04978	Mineral absorption	9.82E-04	TF,ATP1A1
hsa05150	Staphylococcus aureus infection	1.58E-03	C3,C4A
hsa04976	Bile secretion	1.77E-03	CA2,ATP1A1
hsa04512	ECM-receptor interaction	2.49E-03	COL1A2,COL1A1
hsa04970	Salivary secretion	2.71E-03	CALML3,ATP1A1
hsa04972	Pancreatic secretion	3.20E-03	CA2,ATP1A1
hsa04925	Aldosterone synthesis and secretion	3.20E-03	CALML3,ATP1A1
hsa04916	Melanogenesis	3.39E-03	CALML3,TYRP1
hsa04270	Vascular smooth muscle contraction	5.65E-03	CALML3,MYH11
hsa05322	Systemic lupus erythematosus	5.73E-03	C3,C4A
hsa04910	Insulin signaling pathway	6.07E-03	CALML3,FASN
hsa05418	Fluid shear stress and atherosclerosis	6.24E-03	CALML3,GSTA1
hsa01100	Metabolic pathways	6.77E-03	TYRP1,BCAM,FASN,GSTA1,CA2
hsa04261	Adrenergic signaling in cardiomyocytes	7.12E-03	CALML3,ATP1A1
hsa04022	cGMP-PKG signaling pathway	8.84E-03	CALML3,ATP1A1
hsa04530	Tight junction	9.14E-03	MYH11,TUBA1A
hsa05010	Alzheimer disease	9.24E-03	CALML3,APOE

For other genes that are not significantly enriched in the pathway, we can retrieve these genes from the GeneCard database (www.genecards.org/). GeneCard is a searchable, comprehensive and public database containing genetic analysis data that provides concise information on all known and predicted human genes in the genome, proteome, transcription, genetics and function. GeneCard is a comprehensive database of human genes. So the easiest way to see a summary of a gene is to use GeneCard.

As to COAD(Colon adenocarcinoma), LGALS4 is associated with the colon. LGALS4 is a Protein Coding gene, the expression of this gene is restricted to the small intestine, colon, and rectum, and it is under-expressed in colorectal cancer. In the paper (Kim et al., 2013), the authors have demonstrated that LGALS4 is predominantly expressed in the luminal epithelia of the gastrointestinal tract, and its loss of expression plays a key role in colorectal tumorigenesis.

As to GBM(Glioblastoma multiforme), It is a primary brain tumor that develops from astroglial cells. The gene GFAP selected by our method is a protein-coding gene. This gene encodes one of the major intermediate filament proteins of mature astrocytes. It is used as a marker to distinguish astrocytes from other glial cells during development. In the paper (Heiland et al., 2019), the authors demonstrated that tumor associated glial cells are widespread in GBM. In the paper (Tichy et al., 2016), the authors demonstrated that the GFAP gene was over-expression in GBM and that GFAP could be considered as a biomarker of astrocytic pathology in neurological diseases.

As to LUSC(Lung squamous cell carcinoma), The gene SFTPA2 selected by our method is a protein-coding gene. This gene is one of several genes encoding pulmonary-surfactant associated proteins (SFTPA) located on chromosome 10. Mutations in this gene and a highly similar gene located nearby, which affect the highly conserved carbohydrate recognition domain, are associated with idiopathic pulmonary fibrosis. In the paper (Peng et al., 2015), the authors demonstrated that SFTPA2 encodes surfactant protein A that plays a vital role in maintaining normal lung function and has been implicated in various lung diseases, which can accurately distinguished lung cancer from other cancer samples.

As to OV(Ovarian serous cystadenocarcinoma), A product of the MUC1 gene of the genes selected by our method has been used as a marker for different cancers. MUC1 is a Protein Coding gene. In the paper (Hu et al., 2006), the authors demonstrated that MUC1 overexpresses in the majority of ovarian carcinomas and contributes to the metastasis process, promotes tumor formation and metastasis. It plays a role in contributing to ovarian tumor growth.

As to STAD(Stomach adenocarcinoma), The gene PGC selected by our method is a protein-coding gene. The protein encoded by this gene is a digestive enzyme produced in the stomach, Polymorphisms in this gene are associated with susceptibility to gastric cancers. In the paper (Shen et al., 2017), the authors demonstrated that PGC is a comparatively ideal negative marker of gastric cancer.

As to TCHA(Thyroid carcinoma), The S100A6 gene selected by our method is a protein-coding gene. In the paper (Sofiadis et al., 2010), the authors demonstrated that the expression patterns of S100A6 in thyroid carcinoma are unique compared with those of other carcinomas, and over-expression in thyroid carcinoma. S100A6 gene can be used as a biomarker of Thyroid carcinoma.

**Figure 4:**
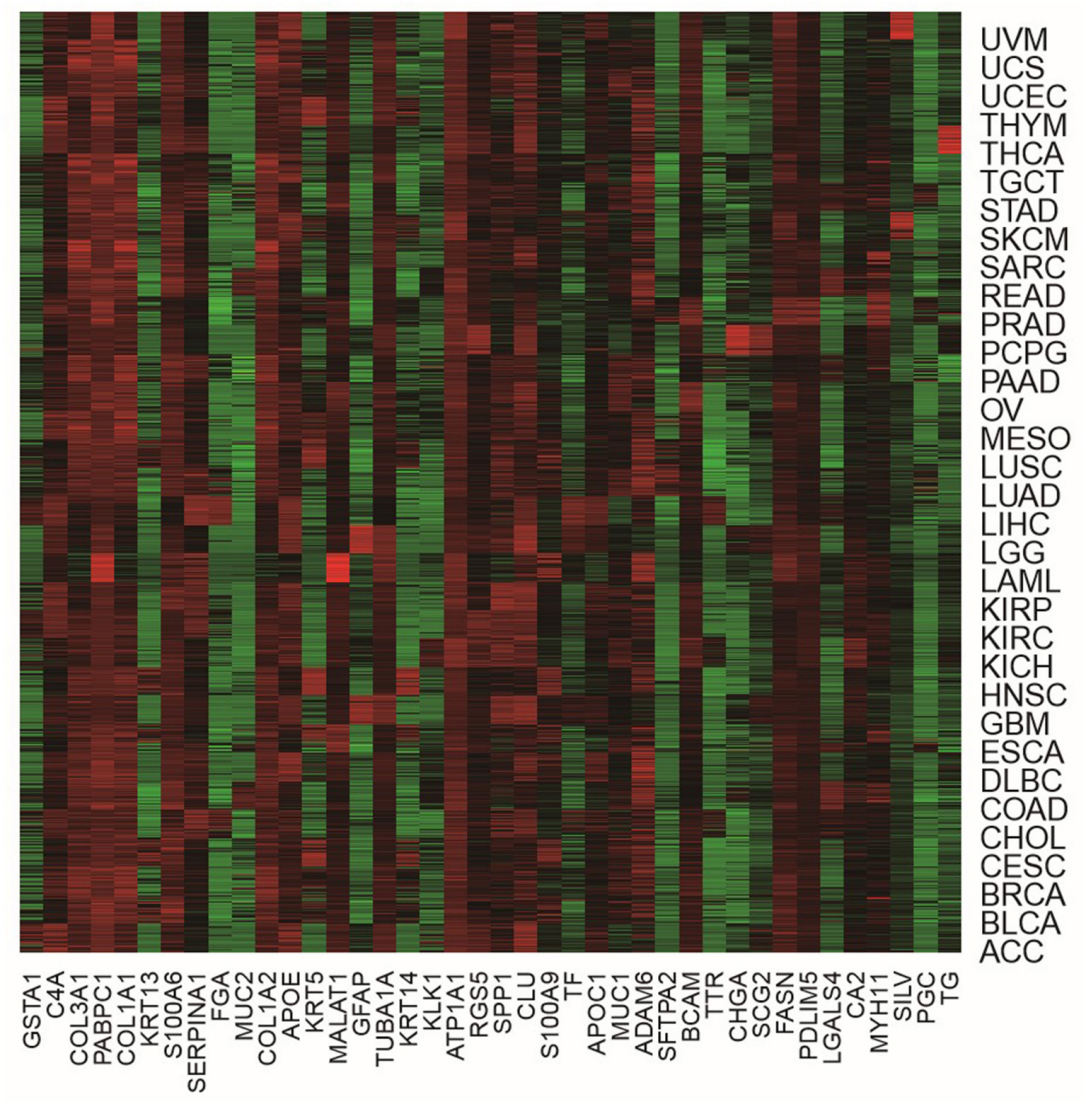
The heat map of the top 40 genes across all tumor samples.

In order to more visually show the expression of genes in different tumor samples, we can use heat maps to understand the distribution of data or the differential expression of genes. In the heat map, the gradient color is used to represent the change of values. The data value size can be visually represented by the defined color depth. In addition, each column represents the expression of each gene in different samples, and each row represents the expression of all genes in each sample. A heat map representation of the relative expression levels of the top 40 genes across all tumor samples is shown in Figure 4.

From Figure 4, we were able to look at the level of expression of each gene in all tumor types. The use of heat maps is more indicative of the relationship between genes and samples. For example, the gene of GFAP was highly expressed in LGG and GBM and low in all other tumors. The gene of LGALS4 was moderately expressed in COAD and READ and low in all other tumors. The heat map visually shows that these genes are differential expression in different tumor samples, which also demonstrates the effectiveness of our proposed method. It is feasible to identify biomarkers with our proposed method.

These results indicate that the genes selected by our method are closely related to the corresponding tumor types, and therefore we can use these selected genes as biomarkers to distinguish different tumors. For the rest of the genes (PABPC1, KRT5, MALAT1, RGS5, SPP1, S100A9, ADAM6, CHGA, SCG2, PDLIM5, SILV), they were neither significantly enriched in the pathway nor found to be tumor-related in GeneCard. The role of these genes in tumor development is unclear, so, pending further study by biological researchers.

## 5. Conclusions and Future Works

In recent years, with the rapid development of the new generation of gene sequencing technology, the generated bioinformatics data such as gene, protein and metabolism are generally high-dimensional and complex. There are a lot of important data closely related to life and health in these data. However, due to the high data dimension, it is impossible to analyze all the data. Feature selection technology can effectively screen high-dimensional data, reduce the workload of data analysis by reducing dimensions, find disease-related markers to achieve early and accurate diagnosis.

In this paper, we have designed a novel approach to classify different types of cancer, whilst it can be used to find biomarkers associated with tumors. We identified biomarkers that were significantly associated with the pan-cancer studies by innovatively combining the traditional machine learning model and deep learning. The presented results and the performance metrics performed in this research showed that the proposed approach achieved an overall testing accuracy of 96.81%. Moreover, the results of our experiment also demonstrated that the genes selected by our method were related to the corresponding tumor types by means of KEGG pathway analysis and gene query, some of these genes have been used as clinical markers. These biomarkers can be used to quickly identify the type of tumor, so as to detect and treat the tumor in advance and improve the cure rate of the tumor.

The methods presented in this paper are not limited to RNA-Seq data, but also applicable to other types of data. However, the method in this paper still needs improvement. For example, the preprocessing strategy of our method includes not only the filter approach, but also the wrapper approach. So, one of the potential future works is applying a new preprocessing strategy to verify and extend this approach. In conclusion, a novel approach for the classification of pan-cancer has been proposed in this paper, which can accurately predict the type of tumor and find tumor-related biomarkers from high-dimensional biological datasets, have broad application prospects and great scientific research prospects, and is of great significance to human development.

## Data Availability Statement

The datasets presented in this study can be found in online repositories. The names of the repository/repositories and accession number(s) can be found in the article/supplementary material.

## Author Contributions

JW and CY conceived and designed the approach. XD performed the experiments. HL and JL analyzed the data. CY and XD wrote the manuscript. JW and GZ supervised the whole study process and revised the manuscript. All authors have read and approved the final version of manuscript.

## Conflict of Interest

The authors declare that the research was conducted in the absence of any commercial or financial relationships that could be construed as a potential conflict of interest.
